# Potential Contributions of Edible Oil and Wheat Flour Fortification on Reducing Inadequate Micronutrient Intake in Ethiopia

**DOI:** 10.1111/nyas.70088

**Published:** 2025-09-17

**Authors:** Kevin Tang, Hiwot Tadesse, Tsedey Moges, Tadesse Kebebe, Gabriel Battcock, Emily Becher, Dawd Gashu, Abel Ahmed, Wendafrash Abera, Saskia de Pee, Masresha Tessema, Frances Knight

**Affiliations:** ^1^ Nutrition and Food Quality Service World Food Programme Rome Italy; ^2^ Department of Population Health London School of Hygiene and Tropical Medicine London UK; ^3^ Ethiopia Country Office World Food Programme Addis Ababa Ethiopia; ^4^ Nutrition, Environmental Health, and Non‐communicable Disease Research Directorate Ethiopian Public Health Institute Addis Ababa Ethiopia; ^5^ Centre for Food Science and Nutrition Addis Ababa University Addis Ababa Ethiopia; ^6^ Food and Beverage Industry Research and Development Center Addis Ababa Ethiopia; ^7^ Ethiopian Food and Drug Authority Addis Ababa Ethiopia; ^8^ Friedman School of Nutrition Science and Policy Tufts University Boston Massachusetts USA

**Keywords:** edible oil, Ethiopia, Household Consumption and Expenditure Survey (HCES), inadequacy, large‐scale food fortification, micronutrient, wheat flour

## Abstract

In 2022, Ethiopia enacted the mandatory fortification of wheat flour and edible oil to counter inadequate micronutrient intake as a risk factor for micronutrient deficiencies. This study aimed to model the potential contributions of fortifying wheat flour and edible oil to reducing the risk of micronutrient inadequacy. The 2015/16 Ethiopian Household Consumption‐Expenditure Survey was used to estimate apparent micronutrient intakes of nine micronutrients and triangulated to existing food consumption and micronutrient surveys. Population risk for inadequate micronutrient intake was assessed overall using a mean adequacy ratio and for individual micronutrients included in the fortification standards. Potential contributions of fortification were assessed by comparing two scenarios across subpopulations: assuming no fortification and full compliance with the fortification policy. The reach of fortifiable wheat flour (39%) and edible oil (70%) suggests that fortifying these vehicles could reduce the risk of inadequate micronutrient intake by 44%, with variation between micronutrients, geographies, urban/rural residence, and socioeconomic status. Even under optimistic fortification scenarios, however, micronutrient gaps would remain for the rural poor. Sustained efforts are needed to drive the implementation of Ethiopia's fortification policy and to coordinate fortification with other interventions targeting populations beyond the reach of fortified foods.

## Graphical abstract

In 2022, Ethiopia mandated wheat flour and edible oil fortification to address micronutrient deficiencies. Using 2015/16 consumption data, this study modeled potential contributions, estimating that fortification could reduce inadequate intake risk by 44%, varying by nutrient, region, and socioeconomic status. However, gaps would persist, especially among the rural poor, highlighting the need for strong policy implementation and complementary interventions to reach underserved populations.

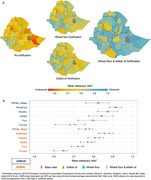

## 1 Introduction

Micronutrient deficiencies (MNDs) pose a significant threat to the health, development, and economic productivity of populations globally [1]. Worldwide, approximately half of preschool‐aged children and two‐thirds of women of reproductive age (WRA) are estimated to be affected by at least one MND [2]. In Ethiopia, the second most populous country in Africa, 50% of adolescent girls and 66% of WRA are deficient in at least one priority micronutrient (i.e., iron, folate, vitamin B12, or vitamin D), as per the most recent micronutrient survey [3, 4]. Multiple risk factors, when combined, can contribute to the manifestation of MND within a population, and need to be addressed together through a diverse combination of interventions (e.g., food fortification, micronutrient supplementation, dietary diversification, agronomic biofortification) enacted through public policy and programs [5, 6]. One key risk factor is inadequate micronutrient intake, where usual intake falls below the respective physiological adequacy window that represents a safe range between inadequacy and excess for the micronutrient [7, 8].

Ethiopia's current strategy for MNDs and all forms of malnutrition has evolved to include nutrition‐sensitive and nutrition‐specific interventions to address inadequate micronutrient intake as part of its broader National Food and Nutrition Strategy (NFNS) [9]. In 2022, Ethiopia enacted the expansion of its mandatory large‐scale food fortification (LSFF) strategy to include the fortification of wheat flour with thiamine, riboflavin, niacin, vitamin B6, folate, vitamin B12, and zinc, and edible oil with vitamin A and vitamin D [10, 11]. As of August 31, 2024, Ethiopia entered the full implementation phase of the LSFF program, marking the end of the grace period and the official launch of the National Food Fortification Program. This launch signals the commencement of mandatory fortification of wheat flour and edible oil according to the new policy, with both the public and private sectors now expected to adopt these fortification standards and ensure compliance. The full implementation plan, codeveloped by the Ministry of Industry and the Ministry of Health [12], provides detailed guidance on strengthening industry capacity, ensuring compliance, and building a robust monitoring and evaluation framework to ensure successful fortification at scale. Implementation of the LSFF standards is underway as a coordinated effort between public, private, and civil societies to improve the effective coverage of LSFF and ultimately provide individuals at greatest risk of MNDs with adequate intakes of micronutrients.

Global guidance recommends program evaluations to assess whether an LSFF program is enabling appropriately fortified products to be available and accessible in sufficient amounts to targeted populations [13]. As Ethiopia progresses into the full implementation phase of the LSFF policy, evidence on how LSFF can be positioned alongside other interventions outlined in the NFNS is critical for assessing the program's potential to contribute to national nutrition goals and identify existing gaps in line with national commitments and global nutrition frameworks [13]. With examples conducted in other country settings, this kind of evidence involves modeling risks for inadequate micronutrient intake without LSFF, estimating the potential LSFF can contribute to meeting micronutrient needs, and identifying additional gaps in program coverage [14, 15].

The diverse existing population survey systems providing insights about nutrition in Ethiopia present a unique opportunity to generate nuanced evidence describing populations potentially at risk of inadequate micronutrient intake and the extent LSFF could support the most nutritionally vulnerable. As part of a formative analysis to assist the implementation of Ethiopia's Food and Nutrition Strategy, the aim of this study is to estimate the potential micronutrient contributions of wheat flour and edible oil fortification using a Household Consumption and Expenditure Survey (HCES) nutrient supply model [16]. More specifically, the study's two objectives seek:

1. To estimate the potential contribution of the mandatory large‐scale fortification of wheat flour and edible oil to reducing risks of inadequate micronutrient intake nationally and for various subpopulations.
2. To triangulate the HCES nutrient supply model with results from existing national food consumption and micronutrient surveys in Ethiopia.

## 2 Materials and Methods

### 2.1 Model Framework and Data Source

The framework for this nutrient supply model has been refined through application across several country contexts and depends on nationally representative HCES [14, 16]. HCES components used within the framework include microdata on household food consumption, household roster information, and characteristics describing subpopulations of interest (e.g., demography, geography, and socioeconomic position).

This study was a secondary data analysis using the 2015/16 Ethiopian Household Consumption‐Expenditure Survey (EHCES) conducted by the Central Statistical Agency of Ethiopia [17]. This EHCES is the fifth round of a repeated cross‐sectional survey that aims to provide data on consumption and expenditure of households to reflect multiple dimensions of poverty. A two‐stage sampling design based on a nationally representative sampling frame derived from the 2007 Population and Housing Census [18] defined 1242 urban enumeration areas (EAs) and 864 rural EAs, where 12−16 households were randomly sampled from each EA. Data collection was conducted between July 2015 and July 2016, and data were collected from 30,229 households in total.

### 2.2 Model Development

The EHCES collected data on household food and beverage consumption using a semi‐open recall conducted from two visits to the household over 1 week. Respondents were asked to recall all foods consumed by members of the household in the previous 3 (visit 1) then 4 (visit 2) days after being prompted with 23 broad food group categories to guide the recall (e.g., cereals, whole grain; pulses, split; meat; vegetables; fruit; etc.). Respondents reported individual food items consumed within each group, quantities consumed converted from nonstandard units, and source (e.g., own production; purchased; etc.) of the food items reportedly consumed. Processing of food consumption data to use for dietary assessment included adjustments for nonedible portions of food, identification and correction of outliers, and transformation to estimate daily consumption.

Micronutrient contributions from the diet, assuming no fortification, were estimated as the base case scenario for risk of inadequate micronutrient intake. To estimate base case micronutrient intakes, each food item reportedly consumed was matched to corresponding values from food composition tables (FCTs) for micronutrients of public health concern for which values were commonly available: vitamin A, thiamine, riboflavin, niacin, vitamin B6, folate, vitamin B12, and zinc. Vitamin D was not included as part of the assessment of risk due to challenges in identifying vitamin D‐rich food items in the EHCES, specifically certain varieties of vitamin D‐rich fish and animal products. The matching process emphasized geographic relevance of the food composition data, where available values from Ethiopian data were prioritized first [19, 20, 21], gaps were filled using the Kenyan FCT second [22], then other international food composition databases last [23, 24]. Micronutrient apparent intake was estimated, where micronutrient contributions from food items were summed to estimate household micronutrient supply, then divided by the number of household members adjusted per adult female equivalent (AFE) [25]. AFEs are derived from the mean daily energy requirement for each household member (parameter assumptions available in Table S1) compared to those of an 18‐ to 29‐year‐old nonpregnant non‐breastfeeding woman, meaning women who are not currently pregnant, breastfeeding, or lactating, for reference [26].

### 2.3 Metrics Used in Assessment of Risk

The primary metric for the assessment of risk for inadequate intake was the mean adequacy ratio (MAR), an indicator of overall nutritional adequacy of a population's diet [27]. This model constructed MARs from household apparent intakes from the EHCES for a group of eight included micronutrients (vitamin A, thiamine, riboflavin, niacin, vitamin B6, folate, vitamin B12, and zinc). Nutrient adequacy ratios (NARs) for each micronutrient were defined as the ratio between the household's apparent intake per AFE and the harmonized average requirement (H‐AR) for an 18‐ to 29‐year‐old nonpregnant, non‐breastfeeding woman [8] on a truncated 0−1 scale. H‐ARs represent the daily micronutrient intake needed by half the healthy individuals in a particular age and sex group and are used as the nutrient reference value when estimating the percentage of the population at risk of apparent inadequacy [7, 13]. NARs represent the proportion of the H‐AR achieved on a 0−1 continuum, which can assess the magnitude of inadequacy relative to the H‐AR [27, 28]. NARs are capped at 1 if the household's apparent intake exceeds the H‐AR, reducing bias from households with high intakes, and preventing these high values from masking the risk of inadequacy of other nutrients when calculating the MAR. MARs were generated as the simple mean of all NARs. Zinc NARs used an H‐AR that assumed a diet high in dietary phytate, based on reported high consumption of unrefined cereal‐based foods.

### 2.4 Objective 1: LSFF Policy Assessment

The reach and consumption of both potentially fortifiable wheat flour and edible oil were assessed. This study assumed that all wheat flour, wheat flour products, and edible oil that were reported as purchased in cash from a market were potentially fortifiable commercially. Reach was defined as the proportion of households in the survey that reported consuming any quantity of each fortification vehicle. Consumption quantity of the population was estimated as the median daily quantity in grams of the fortification vehicle consumed per AFE among households reporting consuming the vehicle. For wheat flour, reach and consumption quantity estimates included reports of wheat flour and wheat flour‐containing products, where quantities from products containing wheat flour were derived using recipe data from FCTs used for the food matches and other relevant literature [29, 30].

The potential of wheat flour and edible oil fortification to contribute to meeting micronutrient shortfalls was assessed. In this full compliance scenario, the quantities of fortifiable vehicles (i.e., wheat flour, wheat flour‐containing products, and edible oil) reportedly consumed by households were assumed to be fortified rather than unfortified. Maximum fortification consumption quantities of 300 g/day per AFE for wheat flour and 100 g/day per AFE for edible oil were used to control for overreporting of vehicle consumption quantities based on estimated portion sizes for each vehicle derived from individual intake data [31]. The fortification specifications as defined by the Ethiopian Standards Agency [10, 11] were used, assuming full compliance, and were adjusted for expected losses using the Food Fortification Formulator Tool [32]. Additional contributions from fortification for each micronutrient were added to the household‐based apparent intake. NARs and MARs were regenerated, accounting for the additional contributions from fortification. Potential additional vitamin D contributions from edible oil fortification were estimated separately using the fortification standards.

### 2.5 Objective 2: Triangulation to Other Food Consumption and Micronutrient Surveys

This study's base case estimated risks of inadequate micronutrient intake from the EHCES were triangulated to two other food consumption and micronutrient surveys: the 2011 Ethiopia National Food Consumption Survey (FCS) [31] and the 2016 Ethiopian National Micronutrient Survey (MNS) [33], using a triangulation framework to classify converging, complementary, or contradicting results between surveys [34]. The classifications are broadly defined as follows: converging results are findings from different surveys that lead to the same conclusion, complementary results are findings from different surveys that lead to differing but supportive rather than contradictory conclusions, and contradicting results are findings from different surveys that lead to opposing conclusions [34]. These definitions informed the study‐specific classification definitions and were tailored to the nature of the data being compared.

First, the three surveys were systematically reviewed to compare survey characteristics and assessment data/methods to consider comparability and identify potential inconsistencies between surveys. Key features included the survey year, sample population, time horizon, sample size, response rate, type of data collected, data collection methods, data processing procedures, and indicators and thresholds.

Next, the EHCES model estimates were triangulated with those from the 2011 FCS, which collected individual‐level intake data from WRA using 24‐hour dietary recall, considered a more precise method for assessing dietary intake for population assessment [35]. The goal of this comparison was to assess consistency in the estimated proportion at risk of inadequate micronutrient intake compared to the prevalence of inadequacy for all included micronutrients. This recognizes that the FCS provides more precise estimates at the individual level for WRA, while the primary use of HCES data is not for dietary assessment and has been known to be subject to bias [36]. For the FCS, observed intake parameters (e.g., food consumption quantities, food composition matches) were used from other studies estimating intake [31, 37], and micronutrient inadequacy was estimated using the Simple Macro tool to derive usual intake distributions and subsequent prevalence of inadequacy using the EAR cut‐point approach [38]. For the EHCES, apparent inadequacy was estimated as the proportion of the population with apparent intake below the H‐AR thresholds used in the previous objective [8]. We classified the comparison between EHCES and FCS estimates using a triangulation framework (Table S6): results were considered *convergent* if EHCES estimates were within 10 percentage points of FCS estimates; and *contradictory* if EHCES estimates were more than 10 percentage points higher than FCS estimates. To further contextualize differences, we compared the median apparent intake from EHCE to the median usual intake from FCS and assessed the contribution of different food groups to total intake.

The dietary intake‐based estimates of risk of inadequacy per AFE from the EHCES and the prevalence of inadequacy from the FCS for children 6−35 months (estimated using the same approach as the estimates of inadequacy for WRA) were triangulated against MND for 6‐ to 59‐month‐old children using biomarker data from the 2016 MNS for vitamin A and zinc. The goal of this comparison was to assess whether the surveys yielded consistent public health problem (PHP) classifications between surveys consistent with international normative guidance. The triangulation framework (Table S6) classified *convergence* when the surveys yielded consistent PHP classifications (e.g., both identifying or both not identifying a PHP). When classifications diverged, the triangulation framework classified the result as *complementary* if the FCS indicated a PHP based on dietary inadequacy, but the MNS biomarker data did not, reflecting situations where intake may be insufficient to meet dietary recommendations but not low enough to manifest as clinical deficiency [39]. For example, if dietary intake data suggested inadequacy at a level that might not yet result in clinical deficiency, this suggested that intake was insufficient to meet dietary needs but not low enough to manifest as a biomarker deficiency. In these cases, dietary inadequacy may precede biomarker‐defined deficiency, providing early warning signals that complement biomarker data even if not fully aligned. Finally, a *contradiction* was classified when the MNS biomarker data indicated a PHP, but the FCS dietary data did not, which is considered as opposing PHP classifications. This divergence could be due to myriad factors, including error, bias, or mismatch in the surveys’ timing, methods, or reference populations, or contributions from other nondietary factors resulting in deficiencies (e.g., infection or other diseases affecting bioavailability or absorption).

PHP classifications based on international normative guidance, and their interpretation for this study, are outlined in Table S7. The World Health Organization (WHO) provides criteria for determining whether vitamin A and zinc deficiencies constitute a PHP based on child population prevalence thresholds from biomarker assessment of deficiencies. For vitamin A, a PHP is defined when the prevalence of low serum retinol (<0.70 µmol/L) is more than 2% for children 6−71 months [40]. Although there are no formal guidelines for classifying a PHP based on dietary intake data alone, WHO ecological criteria allow for supporting evidence from intake data (such as a median dietary intake below 50% of the recommended safe intake among 75% of children 1−6 years) where in this study we defined this classification as the proportion of children from the FCS with intake below the H‐AR exceeding 75%. For zinc, a PHP is defined as the population having a prevalence of low serum zinc (<70 µg/dL) that exceeds 20% [41]. Correspondingly, dietary assessment data suggest that a prevalence of inadequate intake greater than 25% constitutes a potential PHP.

### 2.6 Data Analysis

The reach of fortification vehicles was presented as the percentage of the total population reporting consumption of the fortifiable food, and quantities consumed among consumers were summarized using median consumption quantities and interquartile ranges (IQRs) to assess variance due to a right‐skew distribution. The risk of inadequate micronutrient intake by MAR and NARs was aggregated by subpopulations and summarized using survey means (μ) and 95% confidence intervals (95% CI) to assess variance. The potential contributions LSFF could have toward meeting micronutrient needs were summarized as the percent risk reduction, or the difference in μ between the fortification scenario and the base case relative to the base case (ΔMAR). All population summary estimates were adjusted using household survey weights included as part of the EHCES.

Subpopulations for analysis were classified geographically and socioeconomically. Geographic boundaries used the 2015 national government definitions when the survey was conducted. Reach and consumption of fortification vehicles were presented by regions (administrative level 1), and for the risk of inadequate micronutrient intake, subpopulations were aggregated by region and zone (administrative levels 1 and 2). The risk of inadequate micronutrient intake was presented using maps developed using shapefiles downloaded from GADM (version 4.1) [42]. Socioeconomic positions were defined as urban and rural stratified quintiles of inflation‐adjusted total per capita household expenditure.

Data analysis was conducted using R 4.2.1 in R‐Studio Version 2022.07.1 (RStudio, PBC, Boston, MA). Data management, analysis, and visualization used functions that were available as part of the *tidyverse* package [43]. Population adjustments using survey weights used the functions available as part of the *srvyr* package [44]. Further details about the general data cleaning and processing procedures are published elsewhere [14].

## 3 Results

### 3.1 Description of the Population

From the EHCES sample, 30,218 households had complete records of all necessary framework components and were included in this analysis. The national survey sample had an urban bias requiring survey weight correction, where the sample comprised of 66% urban versus 34% rural households, which deviated from the 16% urban to 84% rural households reported in the census. Subpopulations defined by geographic regions and by religion were generally consistent with the census, and thus representative of the national population distribution. As such, the largest regions by population in the country are Oromia (21%), Amhara (18%), and SNNPR (17%), and the largest religious groups are Orthodox (52%), Muslim (28%), and Protestant (18%). Regions with the highest proportion of households receiving no formal education were Somali (76%), Amhara (69%), and Afar (65%) (Table S3). Households that were poorer and more rural were more likely to have larger household sizes, have lower educational attainment, and depend on skilled, manual, and agricultural work as a primary occupation compared to wealthier and urban counterparts (Table S4).

### 3.2 Objective 1: LSFF Policy Assessment

**FIGURE 1:**
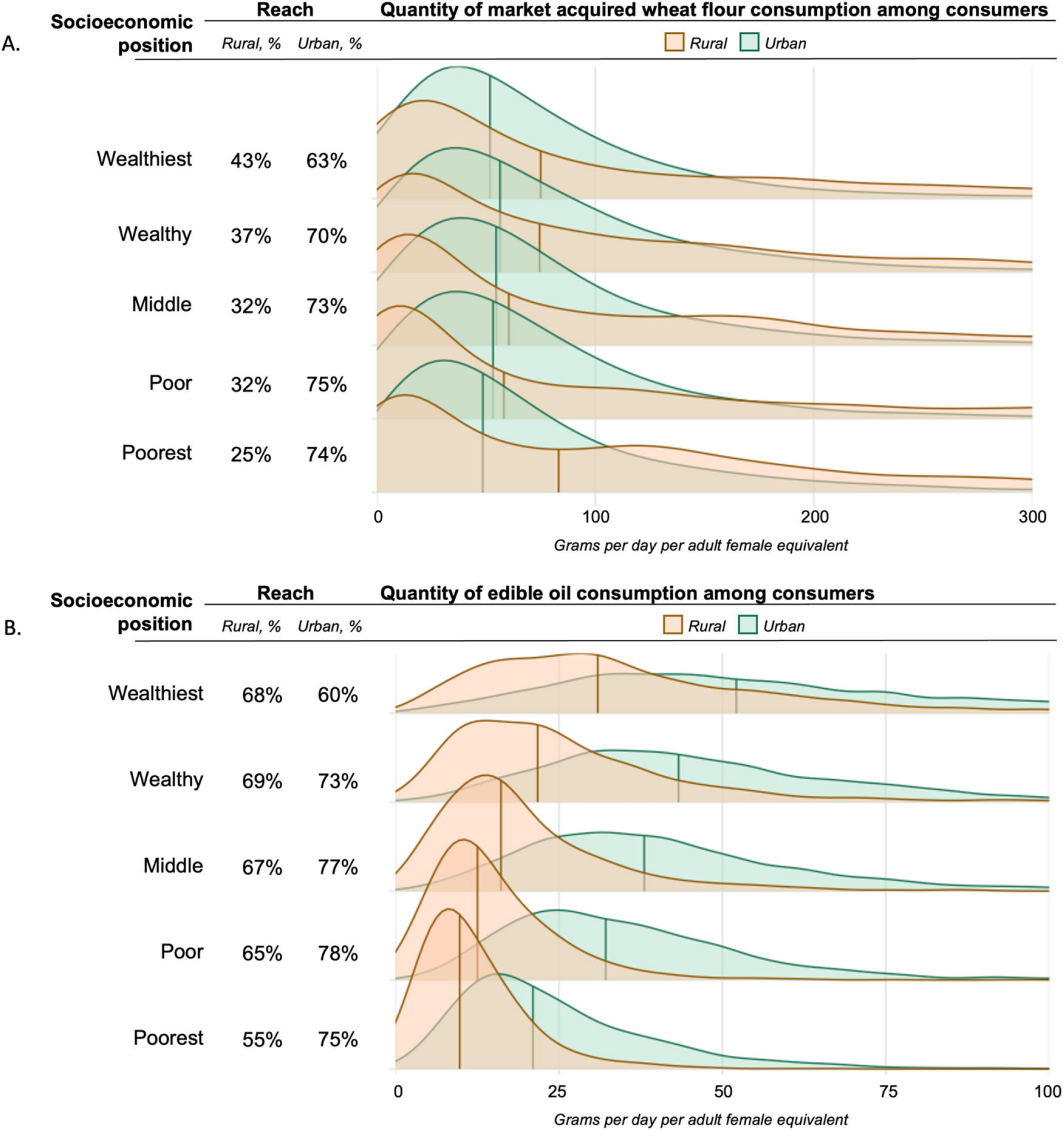
Reach and quantity consumption of fortification vehicles consumed among consumers by socioeconomic position for (A) wheat flour and (B) edible oil.

Table 1 presents the mean base case MAR for the national population and variation by region and by urban‐rural socioeconomic position. Regions with the lowest mean MAR, or at greatest risk of dietary micronutrient inadequacy, were in the east and north of the country: Somali (MAR: μ = 0.63, 95% CI: 0.61, 0.65), Amhara (MAR: μ = 0.64, 95% CI: 0.63, 0.65), Afar (MAR: μ = 0.68, 95% CI: 0.65, 0.70), Benishangul‐Gumuz (MAR: μ = 0.68, 95% CI: 0.66, 0.70), and Tigray (MAR: μ = 0.68, 95% CI: 0.66, 0.70). Rural populations had a marginally lower mean MAR (MAR: μ = 0.72, 95% CI: 0.71, 0.73) compared to urban populations (MAR: μ = 0.76, 95% CI: 0.75, 0.77). In both urban and rural populations, the mean MAR decreased with socioeconomic status, where the poorest populations had the lowest mean MAR and thus the greatest risk of inadequate intake. Figure 1 presents variation in the base case NARs by geographic zone. Micronutrients presenting the lowest risk for inadequate intake (higher NARs) were thiamine, niacin, vitamin B6, and zinc. Micronutrients with a higher risk for inadequate intake (lower NARs) were vitamin A, vitamin B12, folate, and riboflavin. These micronutrients presented a similar geographic distribution to the overall MAR except for vitamin B12, where the largely pastoralist populations in the east of the country presented a lower risk of inadequacy compared to populations in the west.

**TABLE 1:** Base case mean adequacy ratio (MAR) representing the risk of inadequate micronutrient intake and percent difference in MAR when simulating the large‐scale fortification of edible oil and wheat flour.

Population	Sample size, *n* households (%)	MAR (base case), μ (95% CI)	ΔMAR relative to base case		
			Edible oil	Wheat flour	Both vehicles
National	30,218 (100)	0.70 (0.70, 0.71)	27%	23%	44%
Region					
Addis Ababa	3883 (12.7)	0.73 (0.72, 0.74)	34%	23%	54%
Afar	1344 (4.5)	0.68 (0.65, 0.70)	24%	19%	44%
Amhara	5376 (17.8)	0.64 (0.63, 0.65)	27%	28%	43%
Benshangul‐Gumuz	1344 (4.5)	0.68 (0.66, 0.70)	17%	25%	37%
Dire Dawa	672 (2.2)	0.71 (0.69, 0.73)	40%	22%	67%
Gambela	1344 (4.5)	0.79 (0.77, 0.81)	11%	5%	15%
Harari	661 (2.2)	0.74 (0.72, 0.77)	31%	24%	59%
Oromia	6432 (21.3)	0.72 (0.71, 0.73)	24%	24%	43%
SNNPR	5181 (17.1)	0.79 (0.78, 0.80)	18%	6%	27%
Somali	1728 (5.7)	0.63 (0.61, 0.65)	36%	27%	58%
Tigray	2304 (7.6)	0.68 (0.66, 0.70)	21%	19%	35%
Socioeconomic position					
Urban—Total	19,862 (65.7)	0.76 (0.75, 0.77)	28%	22%	49%
Wealthiest	3973 (13.1)	0.88 (0.87, 0.89)	26%	32%	54%
Wealthy	3972 (13.1)	0.82 (0.82, 0.82)	28%	29%	57%
Middle	3972 (13.1)	0.78 (0.78, 0.78)	29%	25%	55%
Poor	3972 (13.1)	0.73 (0.73, 0.73)	29%	21%	51%
Poorest	3973 (13.1)	0.61 (0.60, 0.62)	25%	12%	38%
Rural—Total	10,357 (34.3)	0.72 (0.71, 0.73)	14%	13%	26%
Wealthiest	2074 (6.9)	0.83 (0.82, 0.84)	16%	26%	42%
Wealthy	2073 (6.9)	0.78 (0.77, 0.79)	16%	19%	35%
Middle	2074 (6.9)	0.73 (0.72, 0.74)	15%	14%	28%
Poor	2073 (6.9)	0.68 (0.67, 0.69)	13%	10%	22%
Poorest	2074 (6.9)	0.59 (0.58, 0.60)	12%	6%	17%

**FIGURE 2:**
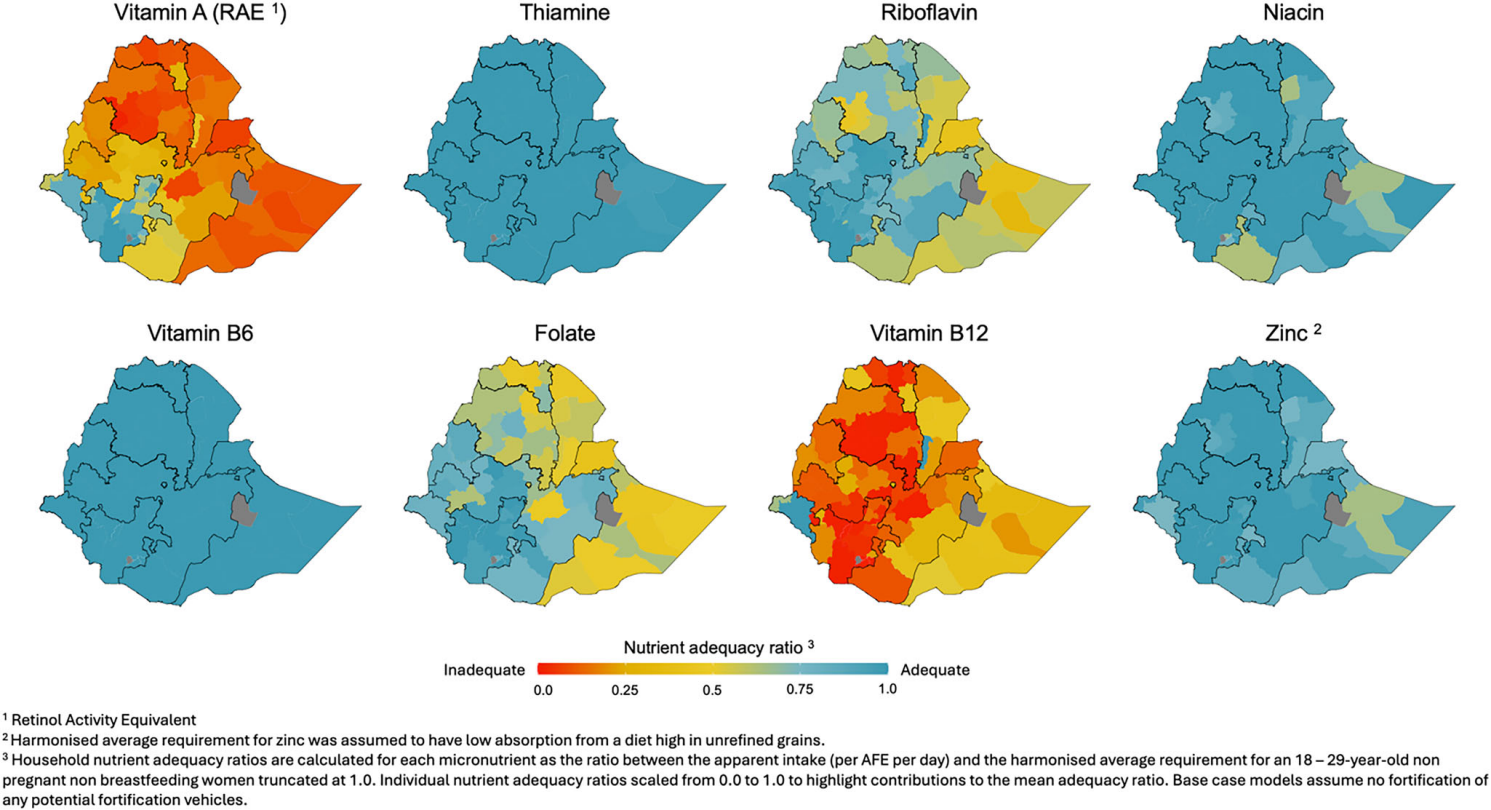
Base case risk of inadequate micronutrient intake from dietary sources for all micronutrients by nutrient adequacy ratios.

Based on the EHCES assessment, the reach of fortifiable wheat flour and edible oil nationally was 39% and 70%, respectively. Fortifiable wheat flour and edible oil consumption by socioeconomic status and urban/rural residence is presented in Figure 2 and by regions in the Supplementary Materials. For wheat flour, reach was higher in urban populations (69%) compared to rural populations (30%), where in rural residences, wheat flour reach was lower for poorer subpopulations. Fortifiable wheat flour was most widely consumed in the country's eastern border (i.e., Somali, Afar, eastern Oromia), the west of the country bordering South Sudan (i.e., Gambela, SNNPR, western Oromia, Benshangul‐Gumuz), and geographies with majority urban populations (i.e., Addis Ababa, Dire Dawa, Harari) (Figure S2). Among consumers, the median quantity of wheat flour consumed was less than 100 g/day per AFE across all subpopulations. For edible oil, reach was higher compared to wheat flour, and with greater similarity of estimates between rural (68%) and urban (74%) residences. Among consumers, the median quantity of edible oil consumed ranged between 10 and 52 g/day per AFE across all populations and was higher in urban residences compared to rural. Furthermore, variation was higher in urban populations (IQR = 30 g/day per AFE) compared to rural populations (IQR = 19 g/day per AFE), where in urban residences, median consumption quantity for the wealthiest quintile (52 g/day per AFE, IQR: 44 g) was greater than the poorest quintile (21 g/day per AFE, IQR: 18 g).

**FIGURE 3:**
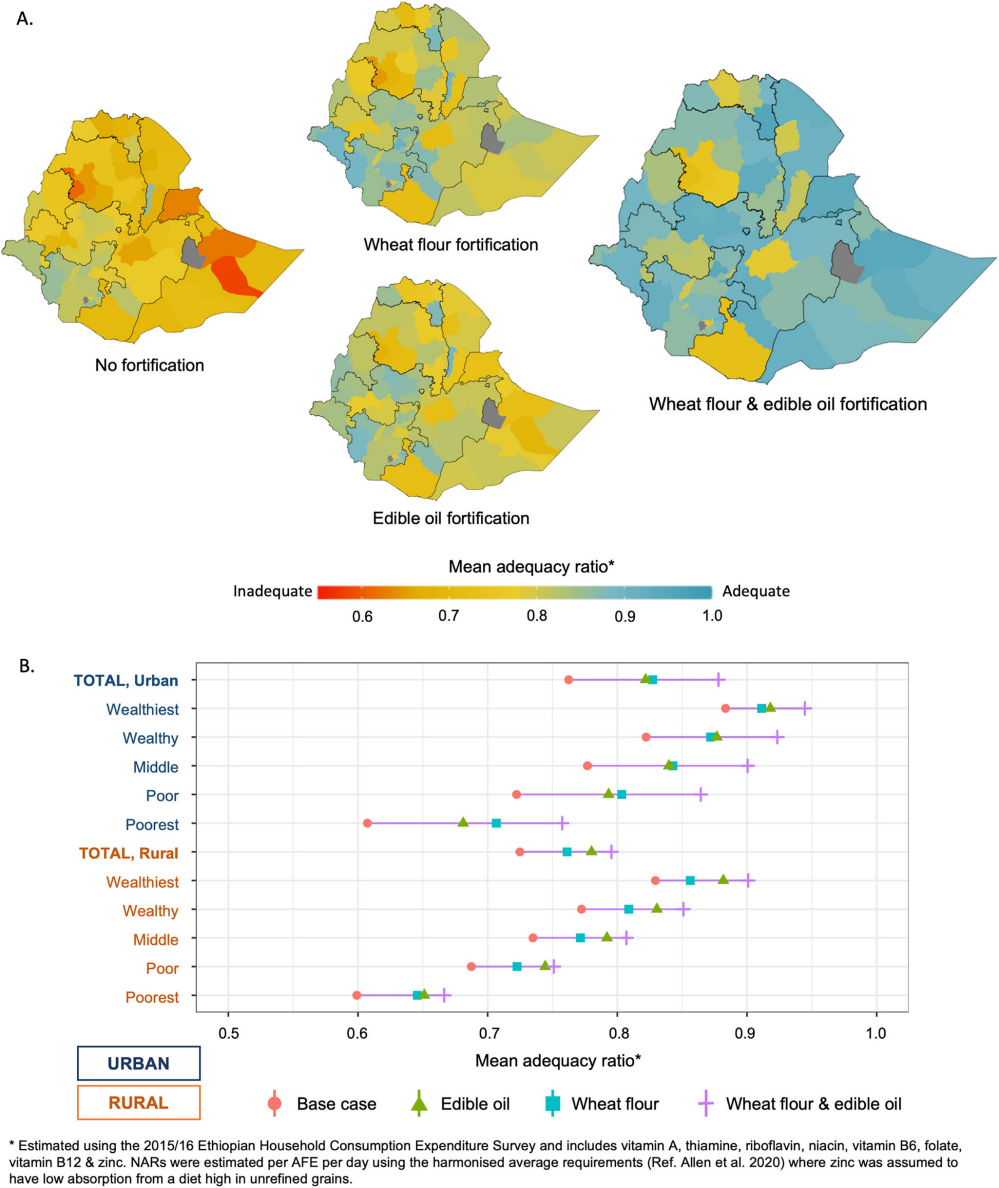
Potential contributions of the wheat flour and edible oil fortification policy on the risk of overall inadequate micronutrient intake by (A) geography and (B) place of residence and socioeconomic position.

The potential that the LSFF policy could contribute to reducing the risk of inadequate micronutrient intake, or ΔMAR, assuming the fortification scenario, is presented in Table 1. Nationally, the risk of inadequate micronutrient intake could be reduced (from the base case) by 23% assuming wheat flour fortification, 27% assuming edible oil fortification, and 44% with both vehicles combined. The regions with the largest potential risk reduction were Dire Dawa (ΔMAR = 67%), Harari (ΔMAR = 59%), and Somali (ΔMAR = 58%), assuming both vehicles were fortified. The ΔMAR by geography, with the addition of each fortification vehicle, is presented visually in Figure 3A. By socioeconomic position, the potential risk reduction from edible oil and wheat flour fortification was lower in rural populations (ΔMAR = 26%) compared to urban populations (ΔMAR = 49%). In addition, the poorest populations in rural residences had the lowest potential reduction in risk, assuming the fortification of both vehicles (ΔMAR = 17%), and the MAR would remain the lowest of all population socioeconomic groups. This indicates that the rural poorest would remain the most disadvantaged, where the initial risk is the highest, but potential contributions from LSFF are lowest. Changes in MAR by socioeconomic status with the addition of each fortification vehicle are presented visually in Figure 3B.

For individual micronutrients, the potential contribution of the LSFF policy for reducing risks of inadequate intake is presented in the Supplementary Materials. Commercial edible oil fortification has the potential to reduce the risk of vitamin A intake inadequacy for much of the population across the country, although this impact may be limited in northern regions (e.g., Tigray, Amhara, and northern Afar) and subpopulations within each region (e.g., rural poorest populations). For micronutrients where the risk of inadequate intake was higher in the east (i.e., riboflavin, folate), wheat flour fortification had the potential to reduce the proportion of the population at risk. For the rural poorest, micronutrients with an NAR less than 0.6, even after accounting for the maximum potential contributions from LSFF, were vitamin A, riboflavin, folate, and vitamin B12. Potential additional vitamin D contributions from edible oil fortification were also lower in rural poor subpopulations compared to other populations (Figure S11).

### 3.3 Objective 2: Triangulation to Other Food Consumption and Micronutrient Surveys

A comparison of survey characteristics across the three surveys used in this study (the EHCES, the FCS, and the MNS) is presented in Table S8. While all three surveys were designed to provide nationally representative population‐level insights, their sampling time horizons varied, potentially introducing differences due to seasonality. The EHCES collected data across a full calendar year (July 2015–July 2016), while the FCS was conducted from June to September 2011, during the lean season, and the MNS was conducted from March to July 2015. When comparing the two dietary surveys, or the EHCES and FCS, there were differences in dietary assessment methods; the EHCES used a 7‐day semi‐open household recall over two visits, whereas the FCS used a 1‐day individual 24‐hour recall, both being consistent with standard methodologies for estimating apparent/usual intake distributions. Additionally, child inclusion criteria differed between the FCS and MNS; the FCS sampled children aged 6–35 months, while the MNS included children aged 6–59 months.

Triangulation between the risk of inadequate intake per AFE from the EHCES and inadequacy of WRA from the FCS is presented in Table S9. Triangulation of the estimates of risk of inadequate intake from the EHCES and inadequacy from the FCS revealed convergence for vitamin A, thiamine, riboflavin, and vitamin B12, where apparent inadequacy estimates from the EHCES were within ±10 percentage points of inadequacy estimates from the FCS. Contradicting results were found for niacin, folate, and zinc, with EHCES estimates consistently lower than those from the FCS by more than 10 percentage points. These contradictions likely reflect known limitations in household‐level dietary assessment methods, which tend to overestimate the consumption of certain food groups. Grains/white roots and tubers had the largest percent difference in contributions to total intake for niacin (26 percentage points higher) and zinc (27 percentage points higher), suggesting an overestimate of items consumed in this food group in the EHCES. Similarly, the share of folate intake derived from animal‐source foods was 37% higher in the EHCES compared to the FCS, suggesting potential bias due to overestimation in reported intake.

Triangulation between the biomarker‐based prevalence of MND from the 2016 MNS and the prevalence of inadequacy from the 2011 FCS for children is presented in Table S10. For vitamin A, both surveys consistently classified the micronutrient as a PHP: the MNS found 14% of children deficient based on serum retinol (11% above the threshold), while FCS estimated the prevalence of inadequacy to be 83% (8% above the threshold). Similar convergence was observed for zinc, where the MNS reported a 92% deficiency prevalence (72% above the threshold), and the FCS reported a 76% prevalence of inadequacy (51% above the threshold).

## 4 Discussion

This study presents the potential contribution that Ethiopia's mandatory fortification policy could have in reducing risks for inadequate micronutrient intake and highlights gaps that may still remain even if full compliance with industry standards were achieved. The estimated risk for inadequate micronutrient intake varied between geographic zones, but the wide reach and combined consumption of fortifiable wheat flour and edible oil suggest that the fortification of these vehicles has the potential to broadly fill micronutrient gaps across the country. Edible oil was consumed across the country and could contribute to reducing the risk of inadequacy for vitamin A and potentially vitamin D. However, the apparent reach of wheat flour was more spatially heterogeneous than edible oil, meaning certain rural populations were less likely to observe the contributions of the additional B‐vitamins and zinc from the fortification policy. Furthermore, even with the model's optimistic assumption that vehicles purchased from the market were fortified in full compliance with the national standards, micronutrient gaps would still remain for the poorest rural populations, specifically for vitamin A, riboflavin, folate, and vitamin B12 at a minimum. The discussion that follows covers what the results suggest about the potential contributions of Ethiopia's LSFF program, the reliability of the EHCES model drawing from this study's triangulation, and short‐ and long‐term considerations for the future positioning of the program within Ethiopia's NFNS.

This study contributes novel evidence to help inform the extent to which LSFF can help Ethiopians achieve micronutrient intake adequacy aligned with Ethiopia's NFNS [9]. If the LSFF program achieved full compliance as per the national standards, the large‐scale fortification of wheat flour and edible oil has the potential to reduce population risk of inadequate intake, especially in urban residences, but gaps would remain for rural poor populations. These gaps in Ethiopia, also observed in other country settings [14, 15], are likely due to heterogeneity in the dietary risk of inadequate intake (i.e., higher risk in the northern, eastern, and poorest population) and limited access to and consumption of fortifiable wheat flour and edible oil as vehicles among some populations (i.e., most limited access in western, rural populations). These gaps were present in this study's optimistic LSFF scenario, even where the broad assumption that all potentially fortifiable vehicles (i.e., those that were purchased) were assumed to meet national standards—a greatly aspirational assumption that may not reflect near‐term realities, at least in the coming years as the new policy is implemented [45]. These findings support the implementation of Ethiopia's LSFF policy as a valuable strategy to reduce the risk of inadequate micronutrient intake, in addition to pursuing broader efforts to advance other components of the NFNS.

Further investigation into the reasons for low access to and consumption of fortifiable vehicles, especially wheat flour, could support program design to promote and facilitate vehicle consumption. Access to fortified foods could be improved via integration within social protection schemes, such as social assistance (i.e., food and cash transfers) or school meal programs [46, 47]. Social protection schemes can serve as an entry point to improve access to fortified foods. However, parallel initiatives are needed to increase the availability of fortified food through open market channels to support program modalities that depend on cash transfers, foster consumer demand, and ensure overall LSFF program sustainability [48]. To ensure the sustainability of Ethiopia's LSFF program, future research should explore not only barriers to access and consumption but also the incentives, market conditions, and regulatory mechanisms that can strengthen the integration of fortified foods into the broader food system. In parallel, sustained advocacy will be critical to drive the implementation of the fortification policy and to align fortification with complementary interventions that reach populations not adequately served through fortified foods alone.

The risk of inadequate intake from the EHCES and estimates of inadequacy from the FCS showed convergence for most micronutrients, suggesting that the EHCES‐based estimates of risk of inadequacy are broadly consistent with a more precise dietary assessment method. For several micronutrients, including vitamin A, thiamine, riboflavin, and vitamin B12, estimates of inadequacy/apparent inadequacy were consistent within ±10 percentage points between the two surveys. However, for some micronutrients, the EHCES model underestimated the prevalence of inadequacy relative to FCS estimates. These discrepancies are complementary with potential limitations in the HCES method: the risk of inadequate zinc and niacin intake, for example, may be underestimated due to overreporting of staple grain consumption in household‐level bulk recall and telescoping (i.e., survey respondents including consumption events that occurred outside the reference period) [49]. Similarly, folate inadequacy may be underestimated due to potential overreporting of dairy products and other animal‐source foods using HCES methods [36]. In addition, the inability of HCES to reflect intrahousehold food allocation patterns may lead to under‐ or overestimation of micronutrient inadequacy for certain individuals within the household [50]. These findings reinforce the need to interpret micronutrient‐specific results from HCES models with caution, especially for micronutrients derived predominantly from food groups with recall challenges. Additionally, some differences between EHCES and FCS estimates may be attributed to variation in survey design, timing, and reference populations: EHCES was conducted in 2015–2016 across all seasons using household‐level recall, while the FCS was conducted in 2011 over 3 months during the lean season using an individual‐level 24‐hour recall method. Future analyses drawing on recent individual‐level dietary intake data [3] could help further validate the accuracy of future EHCES‐based intake models to assess population inadequacy, improve understanding of food group contributions to intake distributions, and support more precise micronutrient‐specific policy recommendations.

Triangulation between dietary inadequacy estimates from the FCS/EHCES and biomarker‐based deficiency data from the 2016 MNS also showed convergence in the classification of vitamin A and zinc as PHPs. This alignment lends further support to the EHCES model's population‐level estimates, particularly for micronutrients where both intake thresholds and biomarker‐based definitions are well‐established (e.g., zinc). Unlike dietary data, which estimate inadequacy or its risks based on intake distributions, biomarker assessments reflect micronutrient status in total body stores and are, therefore, critical for assessing the physiological burden of deficiency in the population [7]. There are several challenges to consider when triangulating dietary and biomarker data, as the two approaches are designed to measure different dimensions of micronutrient status, and are further complicated by differences in survey samples and time horizons. This study's triangulation found converging results, but contradictions are possible. For example, in this study's complementary classification in the triangulation framework, the prevalence of inadequate intake can be a problem even with a low burden of deficiency, when intake is above the level associated with deficiency but still below dietary intake recommendations. This zone of suboptimal intake—above deficiency thresholds but below recommended levels—is important as it signals a micronutrient gap even if not low enough to result in clinical deficiency. The development of theoretical frameworks that explain all possible combinations of results is needed to guide future triangulations of dietary intake and biomarker data (especially for vitamin A [51] and zinc [52]), where multiple biomarkers, comorbidity factors (e.g., inflammation), and bioavailability considerations for dietary assessment [53] all require careful interpretation.

Clearer normative guidance on PHP classification criteria would enable the development and use of triangulation frameworks to characterize micronutrient status. For instance, in the case of folate, there is no internationally accepted population‐level prevalence threshold to define folate deficiency as a PHP [54]. Instead, population red blood cell (RBC) folate concentrations are recommended to be above 400 ng/mL (906 nmol/L) in WRA to achieve the greatest reduction of neutal tube defects (NTDs), but no criteria are provided to define a PHP [54]. A multiperspective assessment of population folate status (e.g., functional outcomes, biomarkers, intake) could be used to assess whether public health action is necessary. For biomarkers, the MNS found that the median RBC folate concentration was 511 nmol/L (IQR: 359, 753), which is below the population threshold of 906 nmol/L for prevention of NTDs. For intake, this study found the risk of folate inadequacy to be 58% (complementing the 81% inadequacy from the FCS). The daily requirements threshold for folate was established based on the prevention of clinical deficiency (i.e., not prevention of NTDs) [55, 56], suggesting that a substantial proportion of the population may not be achieving sufficient intake to protect against both clinical deficiency and functional outcomes like NTDs. Low dietary intake is the primary cause of folate deficiency [55], and lower RBC folate concentrations (and, therefore, lower dietary intake) are associated with a higher risk of NTDs among WRA [57]. While there is currently no formal global threshold to classify folate deficiency or inadequacy as a PHP, the combined evidence of high prevalence of NTDs in Ethiopia [58], low median RBC folate concentrations, and high dietary folate inadequacy indicates that folate deficiency warrants public health attention. This is one example of how multiple sources of data can facilitate triangulation of status for a micronutrient, but similar efforts are needed for other micronutrients of policy relevance where no clear guidance exists.

Ethiopia's national fortification program requires both immediate actions and a long‐term vision to position LSFF as an integrated pillar of the country's NFNS. In the near term, the Ethiopian National Food Fortification Strategic Plan (2023–2027) provides a roadmap for implementation [31]. Immediate action must focus on increasing the effective coverage of fortified foods by improving their availability and affordability in the market, particularly among underserved rural populations. In the longer term, Ethiopia's LSFF program should operate as a dynamic policy instrument that can evolve alongside shifts in food consumption patterns and micronutrient status. Experiences from other countries show the value of periodically adjusting fortification levels and intervention strategies to match the changing dietary consumption patterns and needs of the population [59]. As Ethiopia expands its LSFF program, continued population monitoring and evaluation using future rounds of food and nutrition surveys will be essential to assess whether the current LSFF program design continues to deliver intended benefits.

This study had additional limitations. The use of the 2015/16 EHCES as the primary source of dietary data obstructs this study from generating information specific to priority groups, more specifically, preschool‐aged children and WRA. Additionally, because the data were collected several years ago, they may not fully represent current dietary patterns. While HCES data can provide important perspectives about the general population, insights about how LSFF could contribute specifically to the micronutrient intakes of these populations could be further explored using other large food and nutrition surveys from Ethiopia [3, 4], which collect individual‐level quantitative intake data on these priority groups. Next, this study included just one fortification scenario that assumed full compliance with industry fortification standards. While the aim of this study was to estimate the maximum potential contributions of the policy, this study could be expanded by including scenarios that simulate different stages of the implementation of the policy by integrating monitoring and evaluation data from regulatory entities. Next, the food composition data used to generate the micronutrient apparent intake estimates depended heavily on the Kenyan FCTs due to the limited availability of Ethiopian‐specific food composition data for all micronutrients included at the time of the analysis. The integration of Ethiopian‐specific food composition data, when made available, would improve the geographic relevance of these model parameters. Next, this study assumed that all wheat flour purchased from the market was commercially fortifiable at large scales, which likely overestimated the potential accessibility of wheat flour as a fortification vehicle. Finally, this study used the MAR to summarize micronutrient adequacy across the diet. However, a limitation of this approach is that it does not capture the risk of excessive intake, as NARs are truncated at 1.0 and do not allow for evaluation of intake above the tolerable upper intake level.

## 5 Conclusion

Ethiopia has demonstrated marked progress in defining how LSFF will be integrated into its broader food and nutrition strategy, and this progress must continue as the program matures. This study demonstrates that Ethiopia's large‐scale fortification of wheat flour and edible oil has the potential to substantially reduce population‐level risk of inadequate micronutrient intake, with the greatest benefits observed in urban and wealthier populations. However, even if the LSFF program is operating in full compliance, gaps would still remain for the rural poorest population, requiring innovative approaches to meet needs for these most vulnerable populations. Triangulation with individual intake and biomarker data supports the validity of the EHCES‐based model, while also underscoring the limitations of household‐level dietary assessment for certain nutrients. As Ethiopia's fortification program scales, regular investment in nationally representative dietary and biomarker surveys will be essential to monitor impact. In addition, data on program compliance, equity, and cost‐effectiveness will likely be important for guiding strategic adjustments and ensuring that fortification efforts equitably reach those most in need. With an ambitious vision to see all Ethiopians with “optimal nutritional status, quality of life, productivity, and longevity,” Ethiopia's approach to nutrition ensures that all nutrition interventions, including LSFF, are strategically coordinated to meet the precise nutrition needs for the entire country, especially the most nutritionally vulnerable.

## Author Contributions

KT, HT, FK, and MT set up the collaborative network. KT, HT, TM, TK, and DG designed the study. KT and GB cleaned and transformed the food consumption data. KT and HT compiled the food composition data matches. KT and HT developed the food fortification scenarios. KT, EB, GB, and DG conducted the triangulation. KT, HT, TM, GB, and EB led the interpretation of the results and drafted the manuscript. HT, AA, WA, and MT developed the discussion policy implications. All authors critically reviewed and approved the final manuscript. KT is responsible for the integrity of the data analyzed.

## Conflicts of Interest

There are no potential conflicts of interest relevant to this article.

## Ethical Standards Disclosure

This work is a secondary analysis of deidentified data collected by the Central Statistical Agency of Ethiopia. No Ethics Committee or Institutional Board approval is required.

## Supporting information




**Supplementary Figures and Tables**: nyas70088‐sup‐0001‐SuppMatt.docx

## Data Availability

The household survey data that underlie the base case risk of inadequate intake estimates are hosted by the Central Statistical Agency of Ethiopia and are not publicly available. Food matches and nutrient composition information used in the analysis can be made available upon request from the corresponding author. The R script for the HCES nutrient supply model used to clean, process, and analyze the household survey data is not publicly available because they are actively being used for research and policy engagement in Ethiopia, but it is available on request from the corresponding author.
