# Supplementary material for: Potential Contributions of Edible Oil and Wheat Flour Fortification on Reducing Inadequate Micronutrient Intake in Ethiopia
Source: Ann N Y Acad Sci. 2025 Sep 17;1553(1):270–82. doi: 10.1111/nyas.70088 (PMC12645263; doi:10.1111/nyas.70088)
Supplement: Supplementary file 1 — Supplementary Figures and Tables: nyas70088‐sup‐0001‐SuppMatt.docx [file NYAS-1553-270-s001.docx]

Potential contributions of edible oil and wheat flour fortification on inadequate micronutrient intake in Ethiopia

Supplementary Material

**CONTENTS**

**BACKGROUND**

**Supplementary Table 1.** Parameters and assumptions related to the modelling of the LSFF scenario

**Supplementary Table 2.** Description of the survey population from the Ethiopian Household Consumption-Expenditure Survey.

**Supplementary Table 3.** Description of the survey population by household demographics and education from the Ethiopian Household Consumption Expenditure Survey.

**Supplementary Table 4.** Description of the survey population by household demographics and occupation from the Ethiopian Household Consumption Expenditure Survey.

**OBJECTIVE 1**

**Supplementary Figure 1.** Reach and quantity of fortification vehicles consumed among consumers by geographic region for (A) wheat flour and (B) edible oil.

**Supplementary Figure 2.** Reach of fortification vehicles by geographic region for (A) wheat flour and (B) edible oil.

**Supplementary Table 5.** Mean Adequacy Ratio (MAR) base case and the MAR assuming fortification of edible oil, wheat flour, and both fortifiable vehicles.

**Supplementary Figure 3.** Potential vitamin A contributions from edible oil fortification by (A) geographical zone and (B) residence and socioeconomic position.

**Supplementary Figure 4.** Potential thiamine contributions from wheat flour fortification by (A) geographical zone and (B) residence and socioeconomic position.

**Supplementary Figure 5.** Potential riboflavin contributions from wheat flour fortification by (A) geographical zone and (B) residence and socioeconomic position.

**Supplementary Figure 6.** Potential niacin contributions from wheat flour fortification by (A) geographical zone and (B) residence and socioeconomic position.

**Supplementary Figure 7.** Potential vitamin B6 contributions from wheat flour fortification by (A) geographical zone and (B) residence and socioeconomic position.

**Supplementary Figure 8.** Potential folate contributions from wheat flour fortification by (A) geographical zone and (B) residence and socioeconomic position.

**Supplementary Figure 9.** Potential vitamin B12 contributions from wheat flour fortification by (A) geographical zone and (B) residence and socioeconomic position.

**Supplementary Figure 10.** Potential zinc contributions from wheat flour fortification by (A) geographical zone and (B) residence and socioeconomic position.

**Supplementary Figure 11.** Potential vitamin D contributions from edible oil fortification by residence and socioeconomic position.

**Supplementary Figure 12.** Map of Ethiopia’s regions according to 2015/16 regional boundaries.

**OBJECTIVE 2**

**Supplementary Table 6.** Triangulation framework to compare results from this study’s Ethiopian Household Consumption Expenditure Survey assessment of risk of inadequate intake with the 2011 Ethiopian Food Consumption Survey assessment of inadequacy, and the 2015 Ethiopian Micronutrient Survey assessment of deficiency

**Supplementary Table 7.** Criteria used in this study to define a Public Health Problem (PHP) in accordance with recommendations provided by global guidance

**Supplementary Table 8.** Comparison of the Ethiopian Household Consumption Expenditure Survey characteristics, data, and methods to other surveys included in triangulation.

**Supplementary Table 9.** Triangulation of estimated risk of inadequate micronutrient intake per adult female equivalent from the 2015/16 Ethiopian Household Consumption Expenditure Survey with inadequacy of women of reproductive age from the 2011 Ethiopian Food Consumption Survey (FCS)

**Supplementary Table 10.** Triangulation of estimated risk of inadequate intake from the 2015/16 Ethiopian Household Consumption Expenditure Survey and inadequacy from the 2011 Ethiopian Food Consumption Survey to deficiency from the 2016 Ethiopian Micronutrient Survey for vitamin A and zinc.

**REFERENCES**

**Supplementary Table 1.** Parameters and assumptions related to the modelling of the LSFF scenario

| **Parameters** | | **Data & assumptions** | **Reference(s)** |
| --- | --- | --- | --- |
| *Household micronutrient supply data* | | | |
| Food consumption | | EHCES Data | ^1^ |
| Micronutrient composition of food | | See Supplementary Material | |
| *Adult female equivalent factors* | | | |
| Energy requirement for reference adult female (kcal) | | 2100 | ^2^ |
| Sex of household members | | EHCES Data | ^1^ |
| Age of household members | | EHCES Data | ^1^ |
| Adult male body weight (kg) | | 65 | Assumption |
| Adult female body weight (kg) | | 54 | ^3^ |
| Energy expenditure factor from physical activity  (per basal metabolic rate) | | 1.6 | ^2^ |
| Additional energy requirement for lactation (kcal) | | 500 | ^4^ |
| Energy intake from breastmilk, age 3-5 months (kcal) | | 434 | ^5,6^ |
| Energy intake from breastmilk, age 6-8 months (kcal) | | 413 | ^6^ |
| Energy intake from breastmilk, age 9-11 months (kcal) | | 379 | ^6^ |
| Energy intake from breastmilk, age 12-23 months (kcal) | | 346 | ^6^ |
| *Micronutrient inadequacy thresholds (female 18-50 years)* | | | |
| Vitamin A (μg RAE) | Harmonized Average Requirement | 490 | ^7^ |
| Thiamine (mg) | Harmonized Average Requirement | 0.9 | ^7^ |
| Riboflavin (mg) | Harmonized Average Requirement | 1.3 | ^7^ |
| Niacin (mg) | Harmonized Average Requirement | 11 | ^7^ |
| Vitamin B6 (mg) | Harmonized Average Requirement | 1.3 | ^7^ |
| Folate (μg) | Harmonized Average Requirement | 250 | ^7^ |
| Vitamin B12 (μg) | Harmonized Average Requirement | 2 | ^7^ |
| Zinc (mg) | Harmonized Average Requirement | 10.2 | ^7^ |

EHCES: Ethiopian Household Consumption-Expenditure Survey

**Supplementary Table 2.** Description of the survey population from the Ethiopian Household Consumption-Expenditure Survey (EHCES).

| **Population** | **EHCES 2015/16, n (%)** | **Population & Housing Census 2007, %** |
| --- | --- | --- |
| **Total** | 30,218 (100) | 100 |
| **Region** |  |  |
| Addis Ababa | 3883 (12.7) | 3.7 |
| Afar | 1344 (4.5) | 1.9 |
| Amhara | 5376 (17.8) | 23.3 |
| Benishangul-Gumuz | 1344 (4.5) | 0.9 |
| Dire Dawa | 672 (2.2) | 0.5 |
| Gambela | 1344 (4.5) | 0.4 |
| Harari | 661 (2.2) | 0.2 |
| Oromia | 6432 (21.3) | 36.7 |
| SNNP | 5181 (17.1) | 20.4 |
| Somali | 1728 (5.7) | 6.0 |
| Tigray | 2304 (7.6) | 5.8 |
| **Residence** |  |  |
| Urban | 19,862 (65.7) | 16.1 |
| Wealthiest | 3973 (13.1) | - |
| Wealthy | 3972 (13.1) | - |
| Middle | 3972 (13.1) | - |
| Poor | 3972 (13.1) | - |
| Poorest | 3973 (13.1) | - |
| Rural | 10,357 (34.3) | 83.9 |
| Wealthiest | 2074 (6.9) | - |
| Wealthy | 2073 (6.9) | - |
| Middle | 2074 (6.9) | - |
| Poor | 2073 (6.9) | - |
| Poorest | 2074 (6.9) | - |
| **Religion** |  |  |
| Orthodox | 15,781 (52.2) | 43.5 |
| Muslim | 8423 (27.9) | 33.9 |
| Protestant | 5560 (18.4) | 18.6 |
| Catholic | 200 (0.7) | 0.7 |
| Traditional | 72 (0.2) | 2.6 |
| Other | 102 (0.3) | 0.6 |
| No religion | 78 (0.3) | - |

| Population | Sample size,  n (%) |  |  |  |  |  |  |  |
| --- | --- | --- | --- | --- | --- | --- | --- | --- |
|  |  |  |  |  | Education | | | |
|  |  | Household size, μ | Female headed households, % | Literacy, % | None, % | Primary, % | Secondary+, % | Other/NA, % |
| National | 30,218 (100) | 4.6 | 27 | 46 | 55 | 25 | 15 | 6 |
| Region |  |  |  |  |  |  |  |  |
| Addis Ababa | 3883 (12.7) | 3.9 | 43 | 84 | 17 | 38 | 38 | 6 |
| Afar | 1344 (4.5) | 4.6 | 32 | 36 | 65 | 18 | 13 | 3 |
| Amhara | 5376 (17.8) | 4.1 | 27 | 38 | 69 | 18 | 9 | 4 |
| Benshangul-Gumuz | 1344 (4.5) | 4.4 | 24 | 48 | 49 | 27 | 18 | 5 |
| Dire Dawa | 672 (2.2) | 4.4 | 28 | 62 | 37 | 31 | 28 | 4 |
| Gambella | 1344 (4.5) | 4.6 | 33 | 63 | 30 | 31 | 31 | 8 |
| Harari | 661 (2.2) | 4.0 | 29 | 66 | 32 | 29 | 33 | 6 |
| Oromiya | 6432 (21.3) | 4.9 | 24 | 48 | 50 | 29 | 14 | 6 |
| SNNPR | 5181 (17.1) | 4.9 | 25 | 46 | 48 | 28 | 17 | 7 |
| Somali | 1728 (5.7) | 5.6 | 34 | 29 | 76 | 12 | 9 | 3 |
| Tigray | 2304 (7.6) | 4.4 | 33 | 52 | 56 | 24 | 14 | 6 |
| Socioeconomic position |  |  |  |  |  |  |  |  |
| Urban – Total | 19,862 (65.7) | 3.7 | 37 | 77 | 24 | 33 | 37 | 6 |
| Wealthiest | 3973 (13.1) | 2.4 | 37 | 91 | 9 | 30 | 56 | 5 |
| Wealthy | 3972 (13.1) | 3.1 | 35 | 83 | 17 | 32 | 45 | 6 |
| Middle | 3972 (13.1) | 3.6 | 37 | 78 | 23 | 36 | 35 | 6 |
| Poor | 3972 (13.1) | 4.1 | 40 | 71 | 31 | 33 | 29 | 7 |
| Poorest | 3973 (13.1) | 4.9 | 38 | 61 | 40 | 32 | 21 | 7 |
| Rural – Total | 10,357 (34.3) | 4.9 | 24 | 36 | 65 | 23 | 7 | 5 |
| Wealthiest | 2074 (6.9) | 3.3 | 33 | 44 | 55 | 25 | 14 | 5 |
| Wealthy | 2073 (6.9) | 4.5 | 24 | 38 | 61 | 24 | 9 | 6 |
| Middle | 2074 (6.9) | 5.3 | 22 | 37 | 64 | 24 | 6 | 6 |
| Poor | 2073 (6.9) | 5.4 | 21 | 34 | 67 | 23 | 5 | 5 |
| Poorest | 2074 (6.9) | 6.1 | 17 | 28 | 75 | 18 | 3 | 4 |

**Supplementary Table 3.** Description of the survey population by household demographics and education from the Ethiopian Household Consumption-Expenditure Survey.

**Supplementary Table 4.** Description of the survey population by household demographics and occupation from the Ethiopian Household Consumption-Expenditure Survey.

|  |  |  |  | **Occupations** |  |  |  |
| --- | --- | --- | --- | --- | --- | --- | --- |
| Population | Sample size,  n (%) | Skilled, Manual & Agricultural Workers | High-Level Officials & Professionals | Service & Sales Workers | Elementary & Defense Occupations | Other occupation | Not specified |
| National | 30,218 (100) | 62 | 6 | 7 | 8 | 5 | 11 |
| Region |  |  |  |  |  |  |  |
| Addis Ababa | 3883 (12.7) | 0 | 18 | 22 | 17 | 11 | 29 |
| Afar | 1344 (4.5) | 25 | 8 | 11 | 42 | 3 | 11 |
| Amhara | 5376 (17.8) | 65 | 4 | 6 | 6 | 5 | 12 |
| Benshangul-Gumuz | 1344 (4.5) | 65 | 9 | 5 | 9 | 5 | 7 |
| Dire Dawa | 672 (2.2) | 23 | 14 | 15 | 24 | 8 | 16 |
| Gambella | 1344 (4.5) | 49 | 12 | 12 | 10 | 6 | 8 |
| Harari | 661 (2.2) | 28 | 13 | 20 | 13 | 10 | 13 |
| Oromiya | 6432 (21.3) | 69 | 5 | 6 | 6 | 4 | 9 |
| SNNPR | 5181 (17.1) | 71 | 7 | 6 | 6 | 4 | 6 |
| Somali | 1728 (5.7) | 52 | 4 | 11 | 11 | 1 | 20 |
| Tigray | 2304 (7.6) | 53 | 6 | 8 | 9 | 8 | 15 |
| Socioeconomic position |  |  |  |  |  |  |  |
| Urban – Total | 19,862 (65.7) | 7 | 20 | 22 | 17 | 13 | 20 |
| Wealthiest | 3973 (13.1) | 2 | 37 | 24 | 8 | 12 | 15 |
| Wealthy | 3972 (13.1) | 5 | 25 | 23 | 14 | 13 | 19 |
| Middle | 3972 (13.1) | 7 | 18 | 22 | 19 | 14 | 19 |
| Poor | 3972 (13.1) | 9 | 12 | 21 | 21 | 12 | 23 |
| Poorest | 3973 (13.1) | 14 | 7 | 18 | 25 | 15 | 22 |
| Rural – Total | 10,357 (34.3) | 80 | 2 | 3 | 5 | 2 | 9 |
| Wealthiest | 2074 (6.9) | 66 | 6 | 6 | 5 | 3 | 13 |
| Wealthy | 2073 (6.9) | 80 | 1 | 3 | 4 | 2 | 10 |
| Middle | 2074 (6.9) | 84 | 1 | 2 | 4 | 2 | 7 |
| Poor | 2073 (6.9) | 83 | 1 | 3 | 5 | 2 | 7 |
| Poorest | 2074 (6.9) | 83 | 0 | 2 | 5 | 2 | 7 |

**Supplementary Figure 1.** Reach and quantity of fortification vehicles consumed among consumers by geographic region for (A) wheat flour and (B) edible oil.

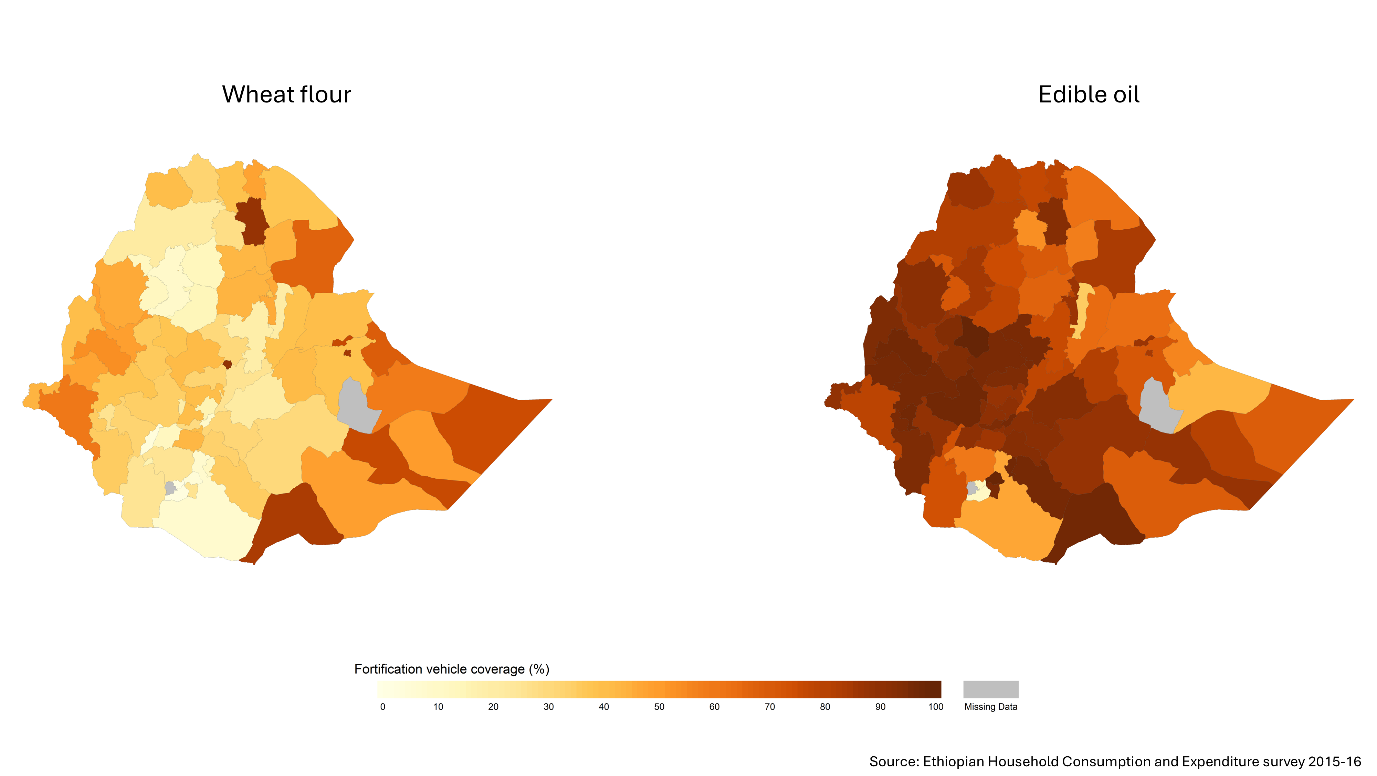


**Supplementary Figure 2.** Reach of fortification vehicles by geographic region for (A) wheat flour and (B) edible oil.

**Supplementary Table 5.** Mean Adequacy Ratio (MAR) base case and the MAR assuming fortification of edible oil, wheat flour, and both fortifiable vehicles.

| Population | Sample size, n households (%) | MAR (base case),  μ | MAR | | |
| --- | --- | --- | --- | --- | --- |
|  |  |  | Edible oil | Wheat flour | Both vehicles |
| National | 30,218 (100) | 0.70 | 0.78 | 0.77 | 0.83 |
| Region |  |  |  |  |  |
| Addis Ababa | 3883 (12.7) | 0.73 | 0.84 | 0.81 | 0.89 |
| Afar | 1344 (4.5) | 0.68 | 0.76 | 0.74 | 0.82 |
| Amhara | 5376 (17.8) | 0.64 | 0.76 | 0.76 | 0.81 |
| Benshangul-Gumuz | 1344 (4.5) | 0.68 | 0.76 | 0.78 | 0.82 |
| Dire Dawa | 672 (2.2) | 0.71 | 0.84 | 0.80 | 0.91 |
| Gambella | 1344 (4.5) | 0.79 | 0.84 | 0.83 | 0.84 |
| Harari | 661 (2.2) | 0.74 | 0.84 | 0.83 | 0.91 |
| Oromiya | 6432 (21.3) | 0.72 | 0.79 | 0.79 | 0.84 |
| SNNPR | 5181 (17.1) | 0.79 | 0.85 | 0.83 | 0.87 |
| Somali | 1728 (5.7) | 0.63 | 0.79 | 0.76 | 0.86 |
| Tigray | 2304 (7.6) | 0.68 | 0.77 | 0.76 | 0.81 |
| Socioeconomic position |  |  |  |  |  |
| Urban – Total | 19,862 (65.7) | 0.76 | 0.83 | 0.81 | 0.88 |
| Wealthiest | 3973 (13.1) | 0.88 | 0.91 | 0.92 | 0.94 |
| Wealthy | 3972 (13.1) | 0.82 | 0.87 | 0.87 | 0.92 |
| Middle | 3972 (13.1) | 0.78 | 0.84 | 0.83 | 0.90 |
| Poor | 3972 (13.1) | 0.73 | 0.80 | 0.78 | 0.86 |
| Poorest | 3973 (13.1) | 0.61 | 0.71 | 0.66 | 0.76 |
| Rural – Total | 10,357 (34.3) | 0.72 | 0.76 | 0.76 | 0.80 |
| Wealthiest | 2074 (6.9) | 0.83 | 0.85 | 0.87 | 0.90 |
| Wealthy | 2073 (6.9) | 0.78 | 0.82 | 0.82 | 0.86 |
| Middle | 2074 (6.9) | 0.73 | 0.77 | 0.76 | 0.80 |
| Poor | 2073 (6.9) | 0.68 | 0.73 | 0.72 | 0.75 |
| Poorest | 2074 (6.9) | 0.59 | 0.64 | 0.62 | 0.66 |

**Supplementary Figure 3.** Potential vitamin A contributions from edible oil fortification by (A) geographical zone and (B) residence and socioeconomic position.

**Supplementary Figure 4.** Potential thiamine contributions from wheat flour fortification by (A) geographical zone and (B) residence and socioeconomic position.

**Supplementary Figure 5.** Potential riboflavin contributions from wheat flour fortification by (A) geographical zone and (B) residence and socioeconomic position.

**Supplementary Figure 6.** Potential niacin contributions from wheat flour fortification by (A) geographical zone and (B) residence and socioeconomic position.

**Supplementary Figure 7.** Potential vitamin B6 contributions from wheat flour fortification by (A) geographical zone and (B) residence and socioeconomic position.

**Supplementary Figure 8.** Potential folate contributions from wheat flour fortification by (A) geographical zone and (B) residence and socioeconomic position.

**Supplementary Figure 9.** Potential vitamin B12 contributions from wheat flour fortification by (A) geographical zone and (B) residence and socioeconomic position.

**Supplementary Figure 10.** Potential zinc contributions from wheat flour fortification by (A) geographical zone and (B) residence and socioeconomic position.

**Supplementary Figure 11.** Potential vitamin D contributions from edible oil fortification by residence and socioeconomic position.


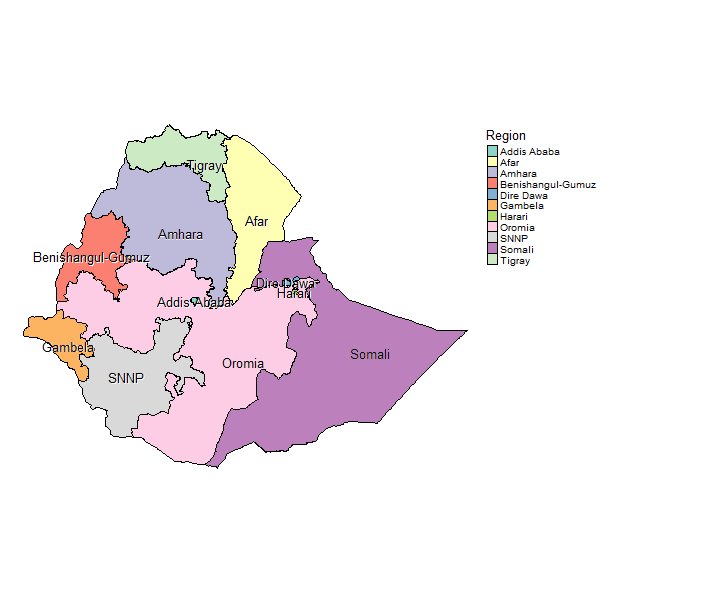


**Supplementary Figure 12.** Map of Ethiopia’s regions according to 2015/16 regional boundaries.

**Supplementary Table 6.** Triangulation framework to compare results from this study’s Ethiopian Household Consumption Expenditure Survey assessment of risk of inadequate intake with the 2011 Ethiopian Food Consumption Survey assessment of inadequacy, and the 2015 Ethiopian Micronutrient Survey assessment of deficiency

|  | **Triangulation classification** | | |
| --- | --- | --- | --- |
| **Triangulation comparison** | Convergent | Complement | Contradicting |
| *Risk of inadequate intake* (EHCES) **vs** *inadequacy* (FCS) for all micronutrients | Risk within ±10% of inadequacy for adult women | - | Risk outside of ±10% of inadequacy for adult women |
| *Inadequacy* (FCS) **vs** *deficiency* (MNS) for vitamin A | Both/neither inadequacy^1^ and/nor deficiency^2^ results indicate a PHP for children. | Inadequacy define PHP, deficiency does not^3^ for children | Deficiency defines PHP, inadequacy does not for children |
| *Inadequacy* (FCS) **vs** *deficiency* (MNS) for zinc | Both/neither inadequacy and/nor deficiency results indicate a PHP for children | Inadequacy define PHP, deficiency does not for children | Deficiency defines PHP, inadequacy does not for children |

1 Assuming that no 4 other criteria indicate a PHP requiring an intervention according to WHO guidelines

2 Prevalence of low serum retinol indicating PHP and at least 3 other demographic/ecological criteria need to be satisfied in order to define PHP requiring an intervention

3 Disregarding potential contributions from supplementation

| Assessment type | Vitamin A | | | Zinc | | |
| --- | --- | --- | --- | --- | --- | --- |
|  | Global guidance PHP | Reference | Study definition PHP | Global guidance PHP | Reference | Study definition PHP |
| Dietary assessment of inadequacy | Median dietary intake lower than 50% of recommended safe level of intake among 75% of children 1-6 years of age.^1^. | [WHO (2011)](https://iris.who.int/bitstream/handle/10665/85859/WHO_NMH_NHD_MNM_11.3_eng.pdf?sequence=4) Serum retinol concentrations for determining the prevalence of vitamin A deficiency in populations. | Prevalence of inadequacy >75% for children 6-35 months, where inadequacy is defined as usual intake below the H-AR | If a country has > 25% of the population at risk of inadequate zinc intakes based on data from the national food supply or from direct assessments of dietary intakes, then country is considered as having an elevated risk of zinc deficiency.  If a country has ≥ 25% of the population at risk of inadequate zinc intakes based on data from the national food supply or from direct assessments of dietary intakes, and ≥ 20% stunting among preschool children, the country should be considered at high risk of zinc deficiency. | [IZiNCG (2004)](https://archive.unu.edu/unupress/food/fnb25-1s-IZiNCG.pdf) Assessment of the risk of zinc deficiency in populations and options for its control | Prevalence of inadequacy >25% for children 6-35 months, where inadequacy is defined as usual intake below the H-AR |
| Biomarker assessment of deficiency | Prevalence of low serum retinol (0.70 µmol/l or below) to define a public health problem and its level of importance among children 6-71 months of age:  2-9% = mild PHP  10-19% = moderate PHP  20%+ = severe PHP | [WHO (2011)](https://iris.who.int/bitstream/handle/10665/85859/WHO_NMH_NHD_MNM_11.3_eng.pdf?sequence=4) Serum retinol concentrations for determining the prevalence of vitamin A deficiency in populations. | Prevalence of low serum retinol >2% for children between 6-59 months | If prevalence of serum zinc below the threshold is >20%, the population is considered as having an elevated risk of deficiency and it is considered a public health concern and national level programs may be considered following further assessment to identify groups at elevated risk. | [IZiNCG (2004)](https://archive.unu.edu/unupress/food/fnb25-1s-IZiNCG.pdf) Assessment of the risk of zinc deficiency in populations and options for its control | Prevalence of low serum zinc >20% for children between 6-59 months |

**Supplementary Table 7.** Criteria used in this study to define a Public Health Problem (PHP) in accordance with recommendations provided by global guidance

1 Currently, the WHO does not have guidelines to define a public health problem according to prevalence of inadequate VA intake in a population. However, prevalence of inadequate VA intake among children under 5 is one of the defined criteria that can be used in conjunction with prevalence of low serum retinol and three other ecological criteria to define a public health problem requiring an intervention (WHO, 2011). The full list of additional ecological criteria are reported in the WHO guidelines.

|  | **EHCES** | **Ethiopian FCS** | **MNS** |
| --- | --- | --- | --- |
| **Survey characteristics** | | | |
| Year | 2015 - 2016 | 2011 | 2015 |
| Survey design | Nationally representative, cross-sectional survey | Nationally representative, cross-sectional survey | Nationally representative cross-sectional survey |
| Sample population | Households (individualised to adult female equivalents) | - Children (6-35 months)  - Adult females (15-45y) | Children (6-59 months) |
| Time horizon | July 2015-July -2016 | June - September 2011  Data collected during the lean season | March – July 2015 |
| Sample size | 30,229 households | 8,079 children | 3,805 households provided consent/ included in study  - 1148 children had serum retinol analysed  - 1143 children had serum zinc analysed |
| Response rate | 99.96% | 98% | 94% |
| **Data & methods** | | | |
| Type of data | Dietary | Dietary | Biomarker |
| Data collection method | Semi-open household recall. Two visits over one week: First asked to recall previous 3 days, second visit recall previous 4 days | Individual 24-hour recall. One-day per participant | Venous blood draw |
| Primary indicator | Risk of inadequate intake, using the EAR-cut point approach on the population apparent intake distribution | Inadequacy, using the EAR-cut point approach on usual intake distributions estimated using external estimates of within-person variation from the Ugandan FCS and the ISU method | Vitamin A: serum retinol concentration using HPCL adjusted for inflammation  Zinc: serum zinc using atomic absorption spectrophotometry adjusted for inflammation |

**Supplementary Table 8.** Comparison of the Ethiopian Household Consumption Expenditure Survey characteristics, data, and methods to other surveys included in triangulation.

EHCES: Ethiopian Household Consumption-Expenditure Survey from 2015/16

FCS: Ethiopia National Food Consumption Survey from 2013

MNS: Ethiopian National Micronutrient Survey from 2015 (Zinc deficiency estimated by Belay et al. 2022)

|  |  |  | **Triangulation classification between EHCES and FCS** |  |  | **Share of intake from food groups, %** | | | | | | | | | | | |
| --- | --- | --- | --- | --- | --- | --- | --- | --- | --- | --- | --- | --- | --- | --- | --- | --- | --- |
| **Nutritional component** | **Survey** | **Inadequacy/ apparent inadequacy** |  | **Intake/ apparent intake** | | Grain/ white roots/ tubers | Pulses | Nuts & seeds | Meat, poultry, & fish | Dairy | Eggs | VA-rich fruits & veg | Dark green leafy veg | Other veg | Other fruits | Other foods |  |
|  |  | % |  | Median | IQR |  |  |  |  |  |  |  |  |  |  |  |  |
| Energy | EHCES | - | - | 2098 | 1590, 2802 | 60.2 | 7.0 | 0.2 | 9.3 | 1.1 | 0.7 | 0.4 | 0.6 | 2.4 | 0.5 | 17.7 |  |
|  | FCS | - |  | 1671 | 1291, 2096 | 46.7 | 7.8 | 4.4 | 6.6 | 10.6 | 5.0 | 1.0 | 2.8 | 1.9 | 2.9 | 10.1 |  |
| Vitamin A | EHCES | 87.3 | Convergent | 101 | 30, 277 | 10.2 | 0.8 | 0.0 | 13.1 | 5.0 | 3.8 | 12.5 | 30.6 | 23.5 | 0.2 | 0.4 |  |
|  | FCS | 81.9 |  | 30 | 5, 180 | 0.4 | 0.5 | 0.0 | 3.8 | 8.3 | 15.7 | 1.9 | 66.8 | 0.2 | 2.9 | 0.1 |  |
| Thiamine | EHCES | 18.9 | Convergent | 1.4 | 1.0, 2.1 | 71.9 | 7.3 | 0.3 | 12.1 | 0.7 | 0.4 | 0.9 | 2.0 | 3.8 | 0.4 | 0.2 |  |
|  | FCS | 25.4 |  | 1.3 | 0.9, 1.7 | 52.3 | 9.6 | 4.6 | 4.6 | 6.5 | 4.7 | 1.3 | 8.3 | 2.2 | 3.8 | 2.0 |  |
| Riboflavin | EHCES | 66.9 | Convergent | 1.0 | 0.7, 1.5 | 51.1 | 8.6 | 0.1 | 15.1 | 6.3 | 2.1 | 1.3 | 7.8 | 7.0 | 0.6 | 0.2 |  |
|  | FCS | 70.2 |  | 1.0 | 0.7, 1.4 | 25.2 | 7.2 | 1.6 | 8.6 | 23.1 | 8.9 | 2.8 | 15.7 | 1.1 | 3.5 | 2.3 |  |
| Niacin | EHCES | 27.5 | Contradictory | 15 | 10.5, 20.8 | 65.5 | 6.4 | 0.4 | 20.7 | 0.2 | 0.0 | 0.8 | 2.3 | 3.0 | 0.4 | 0.2 |  |
|  | FCS | 69.0 |  | 8.6 | 6.0, 12.0 | 39.8 | 6.1 | 5.3 | 21.7 | 5.4 | 1.2 | 3.9 | 9.2 | 1.2 | 3.6 | 2.7 |  |
| Folate | EHCES | 57.9 | Contradictory | 218 | 142, 340 | 37.5 | 6.6 | 0.2 | 38.3 | 0.8 | 1.2 | 1.7 | 5.2 | 7.2 | 1.1 | 0.3 |  |
|  | FCS | 80.8 |  | 149 | 97, 223 | 38.6 | 4.2 | 0.0 | 0.9 | 9.2 | 23.8 | 0.6 | 11.7 | 0.1 | 7.6 | 3.2 |  |
| Vitamin B12 | EHCES | 88.3 | Convergent | 0.07 | 0.0, 0.3 | 2.6 | 0.0 | 0.0 | 82.8 | 9.6 | 5.0 | 0.0 | 0.0 | 0.0 | 0.0 | 0.0 |  |
|  | FCS | 93.3 |  | 0.07 | 0.0, 0.3 | 0.8 | 0.0 | 0.0 | 23.2 | 46.2 | 29.7 | 0.0 | 0.0 | 0.0 | 0.0 | 0.1 |  |
| Zinc | EHCES | 31.3 | Contradictory | 13.1 | 9.2, 18.2 | 60.0 | 11.5 | 0.1 | 21.3 | 1.2 | 0.4 | 0.3 | 2.1 | 2.5 | 0.2 | 0.4 |  |
|  | FCS | 50.4 |  | 7.2 | 4.0, 11.8 | 33.2 | 11.1 | 5.6 | 24.8 | 7.7 | 5.9 | 1.2 | 2.0 | 1.0 | 5.1 | 2.3 |  |

**Supplementary Table 9.** Triangulation of estimated risk of inadequate micronutrient intake per adult female equivalent from the 2015/16 Ethiopian Household Consumption Expenditure Survey with inadequacy of women of reproductive age from the 2011 Ethiopian Food Consumption Survey (FCS)

**Supplementary Table 10.** Triangulation of estimated prevalence of inadequacy for children 6 to 35 months from the 2011 Ethiopian Food Consumption Survey to prevalence of deficiency for children 6 to 59 months from the 2016 Ethiopian Micronutrient Survey for vitamin A and zinc.

| **Micronutrient** | **Inadequacy from FCS** | | **Deficiency from MNS** | | **Triangulation classification between FCS and MNS** |
| --- | --- | --- | --- | --- | --- |
|  | **%** | **PHP** | **%** | **PHP** |  |
| Vitamin A | 83 | Yes | 14 | Yes | Convergent |
| Zinc | 76 | Yes | 92 | Yes | Convergent |

FCS: Ethiopia National Food Consumption Survey from 2011

MNS: Ethiopian National Micronutrient Survey from 2015 (Zinc deficiency estimated by Belay et al. 2022)

PHP: Public health problem as defined by the criteria recommended by the WHO

References

1. Ethiopian Central Statistical Agency. 2018. “*The 2015/16 Ethiopian Household Consumption-Expenditure (HCE) Survey*.” Addis Ababa.

2. FAO/WHO/UNU. 2004. Human energy requirements. Report of a Joint FAO/WHO/UNU Expert Consultation: Rome, 17–24 October 2001. *AO food and nutrition technical report series*.

3. Central Statistical Agency - Ethiopia. 2017. “*Ethiopia Demographic and Health Survey 2016*.” Addis Ababa, Ethiopia: CSA and ICF.

4. US Centers for Disease Control and Prevention. Accessed October 8, 2020. https://www.cdc.gov/breastfeeding/breastfeeding-special-circumstances/diet-and-micronutrients/maternal-diet.html.

5. Walters C.N., H. Rakotomanana, J.J. Komakech, *et al.* 2019. Maternal determinants of optimal breastfeeding and complementary feeding and their association with child undernutrition in Malawi (2015-2016). *BMC Public Health* **19**: 1503. https://doi.org/10.1186/s12889-019-7877-8

6. WHO Programme of Nutrition. 1998. “*Complementary feeding of young children in developing countries: a review of current scientific knowledge*.” Geneva.

7. Allen L.H., A.L. Carriquiry & S.P. Murphy. 2020. Perspective: Proposed Harmonized Nutrient Reference Values for Populations. *Advances in nutrition (Bethesda, Md.)*. https://doi.org/10.1093/advances/nmz096
